# *PSMC2* is Up-regulated in Pancreatic Cancer and Promotes Cancer Cell Proliferation and Inhibits Apoptosis

**DOI:** 10.7150/jca.27616

**Published:** 2019-08-27

**Authors:** Jing Qin, Wangsheng Wang, Fangmei An, Wei Huang, Junli Ding

**Affiliations:** 1 Department of Medical Oncology, Zhejiang Cancer Hospital, Hangzhou, 310022, P.R. China; 2 Department of Oncology, Wuxi Hospital of traditional Chinese Medicine, Wuxi, 214071, P.R. China.; 3 Department of Gastroenterology, Wuxi People's Hospital, Affiliated to Nanjing Medical University, Wuxi, 214023, P.R. China.; 4 Department of Oncology, Wuxi People's Hospital, Affiliated to Nanjing Medical University, Wuxi, 214023, P.R. China.

**Keywords:** pancreatic cancer, PSMC2, the 26S proteasome, proliferation, apoptosis

## Abstract

**Objective**: The lack of effective therapeutic targets poses a leading challenge to prolong survival and improve the quality of life for pancreatic cancer patients. Proteasome 26S subunit ATPase 2 (*PSMC2*), a recently discovered gene, has been implicated in certain human carcinogenesis. However, limited data is available concerning the functional significance of *PSMC2* in cancer cell growth, and whether it plays a role in pancreatic carcinogenesis remains unknown.

**Materials and Methods**: Quantitative RT-PCR (qRT-PCR) was performed to assess mRNA expression levels of *PSMC2* in different pancreatic cancer cell lines. Knockdown of *PSMC2* was achieved by using short hairpin RNA (shRNA). The effects of *PSMC2* silencing on pancreatic cancer cell proliferation and apoptosis were evaluated by the MTT cell proliferation assay, Celigoassays, Annexin V fluorescence-activated cell sorting (FACS) assay and Caspase-3/7 array.

**Results**: High expression of *PSMC2* was detected in three pancreatic cancer cell lines (SW1990, PANC-1, and AsPC-1). Knockdown of *PSMC2* in SW1990 cells inhibited proliferation and enhanced apoptosis.

**Conclusions**: Our primary study suggests that *PSMC2* might be involved in the progression of pancreatic cancer and may serve as a potential therapeutic target.

## 1. Introduction

Pancreatic adenocarcinoma remains one of the most rapidly fatal human malignancies 1.Owing to the lack of effective methods of early diagnosis and a high probability of invasion and metastasis, pancreatic adenocarcinoma still has the worst prognosis with a median overall survival(OS) of 6 months and a 5-year survival rate of less than 5% 2. Therefore, there is an urgency to discover potential novel targets and effective agents to improve the prognosis of pancreatic adenocarcinoma patients.

The 26S proteasome is responsible for protein degradation in the eukaryotic cells. It consists of a 20S core catalytic subunit and a 19S regulatory subunit. The target protein substrate with a polyubiquitin-tag is first recognized by 19S regulatory subunit, and then unfolded and translocated into 20S core subunit for degradation 3. Either one or two 19S regulatory complexes combine with a single 20S catalytic complex to form, respectively, a singly or doubly capped (26S1 or 26S2) complete 26S proteasome 4. By specifically eliminating target proteins, the 26S proteasome is involved in almost every biological activity, such as cell division, angiogenesis, immune response, transcription factor activation and post-translational modification of proteins. Given its importance, the 26S proteasome is a multifaceted target for anti-cancer therapies 5. Indeed, targeting the 26S proteasome has been proved successful in the treatment of aggressive hematopoietic tumors 6.

Proteasome 26S subunit ATPase 2 (*PSMC2*), a newly identified gene on chromosome 7q22.1-q22.3, encodes an essential member of the 19S proteasome 7, 8. It is essential for 19S and 26S proteasome assembly 9. Nijhawan *et al*. ranked PSMC2 as a top gene in copy number alterations yielding cancer liabilities owing to partial loss (CYCLOPS), which represents a distinct class of cancer-specific liabilities resulting from genome instability and is related to cell proliferation or survival. In ovarian cancer cells, reduction of *PSMC2* expression inhibited cell proliferation. In the same study, *PSMC2* expression also reportedly correlated with pancreatic cancer cell proliferation. However, no further studies had been carried out 8. Recently, Song *et al*. showed high levels of PSMC2 in osteosarcoma samples by tissue microarray analysis and that down-regulation of PSMC2 suppressed osteosarcoma cell proliferation and enhanced apoptosis 10. In addition, *PSMC2* was identified to have synthetic lethal interaction with alveolar soft-part sarcoma-associated *ASPSCR1* in human cells 11. Nevertheless, functional validation and mechanistic studies for PSMC2 in cancers have been lacking. Here, we report, for the first time, the effects of *PSMC2* on pancreatic cancer cell proliferation and apoptosis.

## 2. Materials and Methods

### 2.1 Patients and tumor samples

In this study, stage I-III, formalin-fixed, paraffin-embedded blocks of tumor tissues were obtained from 40 patients with pancreatic adenocarcinoma. All included patients underwent surgical treatment at Wuxi People's Hospital, an affiliate of Nanjing Medical University (Wuxi, China), between January 2013 and December 2015. Patients were enrolled in this study based on the following selection criteria: i) histologically confirmed pancreatic adenocarcinoma other than adenosquamous carcinomas, neuroendocrine carcinomas, mixed subtypes and mucinous neoplasms with associated invasive carcinoma, ii) complete resection performed, iii), sufficient surgically resected tissue was available for formalin-fixed paraffin-embedding. All patients underwent macroscopic curative resection by total pancreatectomy, pancreaticoduodenectomy, or pylorus preserving pancreaticoduodenectomy with lymph node dissection. All resected primary tumors and lymph nodes were examined histologically by hematoxylin and eosin staining according to the TNM classification system 12. This study was conducted in accordance with recognized ethical guidelines (Declaration of Helsinki) and was approved by the Regional Ethics Committee of the hospital. The written informed consents were from the patients or their living relatives.

### 2.2 Immunohistochemistry (IHC)

Tissues were fixed in 10% formalin, embedded in paraffin, and dehydrated in 70% ethanol. Five-μm-thick sections were de-paraffinized in xylene, rehydrated in graded ethanol, and subjected to antigen retrieval by steam heating in Citra ™ antigen retrieval solution (BioGenex). PSMC2 primary antibody (Biorbyt Ltd, Cambridge, Cambridgeshire, UK) was applied to tissues at 1:100. It was defined as positive in the presence of ≥ 5% of neoplastic cells, as previously described 10. It was based on the percentage of neoplastic cells stained positive for PSMC2, with (-) denoting 0.0-5.0% of osteosarcoma cells stained, (+) denoting 5.0-30.0% of osteosarcoma cells stained, (++) denoting 31.0-50.0% of osteosarcoma cells stained and (+++) denoting 51.0-80.0% of osteosarcoma cells stained.

### 2.3 Oncomine database analysis

Oncomine database (https://www.oncomine.org/resource/login.html), an online database consisting of previously published and open-access microarray data, was performed to identify the transcription level of *PSMC2* gene in pancreatic adenocarcinoma 12. The mRNA expression of *PSMC2* in clinical pancreatic adenocarcinoma tissue was compared with that in normal control, using a Student's *t*-test to generate a *p* value. The parameters *p*-value < IE-4, fold change >2, and gene ranking in the top 10% were used to obtain the most significant* PSMC2* probes.

### 2.4 Cell lines and cell culture

Three pancreatic cancer cell lines (SW1990, PANC-1 and AsPC-1 cells) were obtained from American Type Culture Collection (Manassas, VA) and cultured in Dulbecco's modified Eagle's medium (DMEM; Gibco BRL Co. Ltd. USA) supplemented with 10% fetal bovine serum(FBS; Gibco), 100U/ml penicillin, and 100mg/ml streptomycin. Cells were cultured in a standard humidified incubator at 37°C in a 5% CO_2_ atmosphere.

### 2.5 Lentivirus transduction

Small interference RNA (siRNA) was designed to target the human *PSMC2* gene (Gene ID, 5701). The siRNA sequence is as follows: si-*PSMC2*: 5-GCCAGGGAGATTGGATAGAAA-3. Lentivirus without siRNA insert was used as a control. SiRNA constructs were generated by synthesizing and cloning into the pGCSIL-green fluorescent protein (GFP) plasmid vector GV115 with Age I/EcoRI sites (GeneChem, Shanghai, China). Lentivirus was packaged into 293 T cells using virus titersand Lipofectamine 2000 (Invitrogen, Carlsbad, CA, USA) as soon as cell density reached 70%. The interference efficiency of *PSMC2*-siRNA in 293 T cells was tested by GFP. On the third day after transfection, we collected lentivirus particles expressing *PSMC2*-siRNA from cell culture medium. Lentivirus was concentrated using the centrifugal ultrafiltration device (Millipore, Billerica, MA, USA), and then stored at -80°C until use. PANC-1 cells, at 30% confluence in 6-well plates, were infected with a suitable quantity of lentivirus containing *PSMC2*-siRNA or non-targeting siRNA at 37°C in the presence of 6μg/ml polybrene (Sigma-Aldrich, St Louis, MO, USA). Construction of recombinant lentivirus (LV-PSMC2 (19674-1)) containing *PSMC2* (GeneChem, Shanghai, China) was also accomplished by using similar methods as above. After lentiviral construction, PANC-1 cells were transfected with lentivirus to generate *PSMC2*-overexpressing pancreatic cancer cells. Cells transfected with the empty GFP lentivirus were selected as negative controls.

### 2.6 Quantitative RT-PCR (qRT-PCR)

Total RNA was extracted from cells using TrizolReagent (Invitrogen, Carlsbad, CA, USA) according to the manufacturer's protocol. Quantity and quality of the extracted RNA were analyzed by spectrophotometer (Nanodrop2000/2000c; Thermo Fisher Scientific, Inc., Wilmington, DE). mRNA (2ug) was reverse-transcribed using M-MLV Reverse Transcriptase (M1705,Promega Corp., Madison, WI) to synthesize cDNA. QRT-PCR was performed using SYBRVR Premix Ex TaqTM (Takara Bio Inc., Dalian, China) and Mx3000P QPCR System (Agilentn Technologies, Santa Clara, CA). Glyceraldehyde 3-phosphatedehydrogenase (*GAPDH*) was used as an internal control. PCR conditions were strictly controlled as follows: ten minutes at 95°C followed by 40 cycles of 15 seconds at 95°C and 60 seconds at 60°C. After normalization with controls, fold changes of mRNA levels were calculated via the Δ ΔCt method.

### 2.7 Western blot analysis

Protein extraction from cells was performed using radioimmunoprecipitation assay (RIPA) buffer. Protein concentrations were determined using the BCA method (P0010S; Beyotime Institute of Biotechnology). Equal amount of protein lysates were electrophoresed by 10% SDS-PAGE and transferred to the polyvinylidene difluoride (PVDF) membranes (Millipore, Billerica, MA). The membranes were blocked by TBST (NaCl 500mM, Tris 20 mM, pH 7.5) containing 5% skim milk. Protein expression in tissue samples was probed with rabbit polyclonal anti-PSMC2 (sigma, F1804) and mouse anti-GAPDH (Santa Cruz Biotechnology, CA).Mouse anti-FLAG and anti-GAPDH antibodies (Santa Cruz Biotechnology, CA) were used for analyzing protein expression in SW1990 cells exogenously transfected with a plasmid encoding flag-tagged PSMC2. Blots were visualized with Electro Chemical Luminescence system, and densitometric analysis was measured with image analysis system (Thermo Fisher Scientific, MA). Values for the PSMC2 bands were normalized relative to the GAPDH bands.

### 2.8 Cell growth assay determined by Celigo imaging cytometer

Celigo Imaging Cytometer (Nexcelom Bioscience, Lawrence, MA) was used to count the number of surviving cells. Seventy-two hours after infection, cells were collected and seeded into 96-well plates at the density of 1500 cells per well. Cell growth curves and fluorescent photomicrographs were taken by measuring cells with green fluorescence with the cytometer in the following 5 days.

### 2.9 Cell apoptosis analysis

Apoptotic cells were measured using the Annexin V apoptosis kit according to the manufacturer's instructions (88-8007, eBioscience). Briefly, cells were trypsinized, washed once with PBS, and centrifugated at 1300 rpm for 5 min. After that, cells were washed once in 1×binding buffer and resuspended in 1×staining buffer to 10^6^-10^7^cells/ml. Then, 100μl cell (about 5×10^5^ cells) were incubated with 10μlAnnexin V-APC at room temperature for 15 min in the dark. Finally, annexin V-stained cells were analyzed with flow cytometer (Millipore, Billerica, MA) as a measure of cell apoptosis.

### 2.10 MTT cell proliferation assay

The proliferation viability of SW1990 cells transfected with *PSMC2*-lentivirus and control lentivirus vectors were measured using MTT assays. Briefly, cells were seeded in 96-well plates at a seeding density of 2000 cells per well. After 1, 2, 3, 4 or 5 days, a total of 20μl MTT solution (5 mg/ml) were added to the cells for 4h. Cell supernatants were then removed and each well was treated with 100μl dimethyl sulfoxide. After 5 min, the optical density (OD) at 490nm was measured by a microplate reader.

### 2.11 Caspase-3/7 array system

The activity of caspase-3/7, central effector caspases in apoptosis, was measured in SW1990 cells transfected with *PSMC2*-lentivirusand control lentivirus vectors by using Caspase3/7 assays (G8091, Promega). Briefly, infected cells were seeded in a 96-well plate at a density of 1×10^4^cells per well. After addition of equal volume (100μl) Caspase-Glo 3/7 reagent, each well was constantly shaken for 2h at ambient temperature. Luminescence was measured by using a Tecan Infinite M2009 PR plate reader.

### 2.12 Statistical analysis

Statistical analysis was performed using SPSS 17.0 statistical software (SPSS Inc., Chicago, IL, USA). All data were presented as mean ± SD. Each experiment was done in triplicates. Student's *t*-test, Fisher's exact test and one-way analysis of variance were used to analyze and determine statistical significance. *P*<0.05 was considered as significant.

## 3. Results

### 3.1 The high expression of PSMC2 protein in pancreatic adenocarcinoma

**Figure 1:**
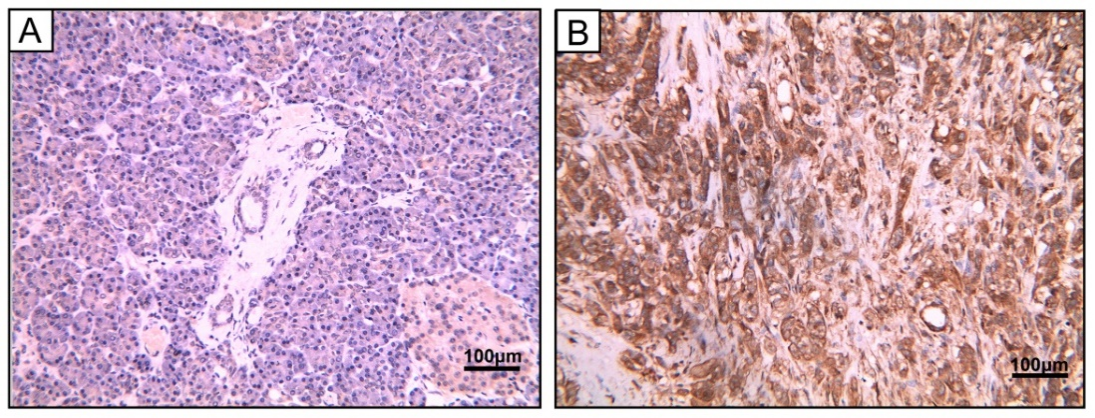
PSMC2 expression in pancreatic adenocarcinoma samples. Representative image of pancreatic adenocarcinoma tissue. (A) Hematoxylin and eosin (H&E)-staining (B) PSMC2 positive expression by IHC. Magnification, 400×.

To study the protein expression of PSMC2 in pancreatic adenocarcinoma, 40 pancreatic adenocarcinoma samples and 5 chronic pancreatitis samples were detected by IHC. Of the 40 tumors evaluated in this study, 28 (70%) were strongly cytoplasm positive for PSMC2 expression in cancer cells, while it was negative in all the chronic pancreatitis tissues. The detailed clinical information and the correlation between PSMC2 expression and clinicopathological characteristics were indicated in Table 1, and Figure 1 showed the representative images. The results suggested that PSMC2 expression was significantly associated with vascular invasion (*P*=0.014) and lymphatic invasion (*P*=0.008).

### 3.2 The transcript expression of PSMC2 in pancreatic adenocarcinoma

**Figure 2:**
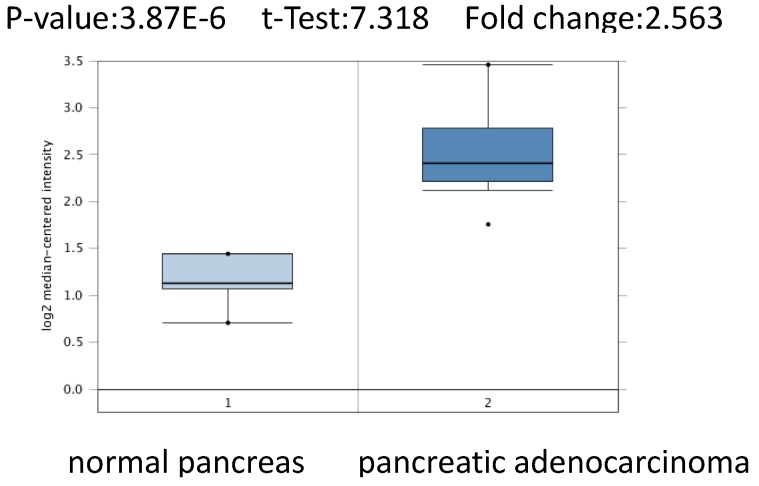
*PSMC2* analysis in pancreatic adenocarcinoma (Oncomine database). The box plot comparing specific PSMC2 expression in normal (left plot) and pancreatic adenocarcinomatissue (right plot) was derived from Oncomine database. The analysis was shown in pancreatic adenocarcinoma relative to normal pancreas.

To further confirm the role of PSMC2 in pancreatic adenocarcinoma, we compared the transcription levels of PSMC2 in pancreatic adenocarcinoma tissues with that in normal tissues, using Oncomine database and found that the mRNA expressions of *PSMC2* were significantly over-expressed in carcinoma tissues as compared to the normal sample. As show in Figure 2, PSMC2 may work oncogenic function.

We conducted cDNA microarray analysis by using the Oncomine database to explore gene expression of *PSMC2* in pancreatic adenocarcinoma. The Oncomine database was queried for PSMC2 expression in pancreatic adenocarcinoma tissues and normal tissues 13. Our analysis revealed that *PSMC2* was over-expressed in pancreatic adenocarcinoma, as compared to that in normal tissue (Figure 2).

### 3.3 Expression of *PSMC2* in pancreatic cancer cell lines

**Figure 3:**
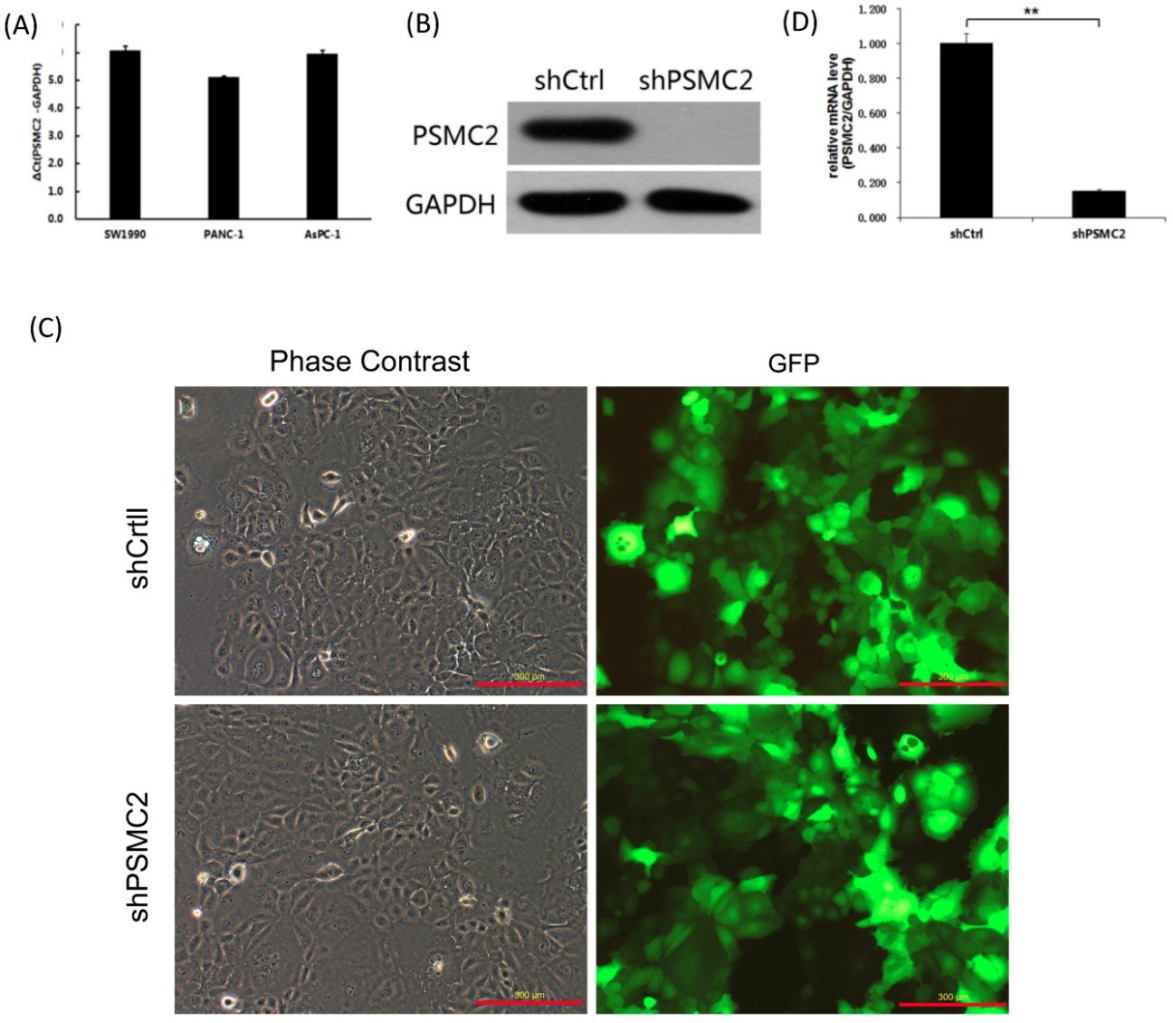
*PSMC2* expression was effectively knockdown by shRNA. (A), *PSMC2* expression in pancreatic cancer cell lines, as determined by qRT-PCR. (B), Efficiency of *PSMC2* silencing in SW1990 cells, as measured by Western blot. (C), Infection efficiency of SW1990 cells with shRNA or shCtrl lentiviral vectors. Cells were assessed by fluorescent microscopy and light microscopy at day 3 post-infection. It is apparent that more than 80% of cells expressed GFP. Magnification, x100. Representative images of the cultures are shown. (**p<0.01). (D), *PSMC2* knockdown in SW1990 cells. Levels of *PSMC2* in SW1990 cells infected with shRNA or shCtrl lentivirus were determined by using qRT-PCR at day 5 post infection. Note that *PSMC2*mRNA level was efficiently down-regulated after shRNA infection.

We first assessed *PSMC2* mRNA levels in a panel of different pancreatic cancer cell lines (SW1990, PANC-1 and AsPC-1) using qRT-PCR. Currently, we had no immortalized pancreatic epithelial cell lines. Postoperative specimens of 3 pancreatic cancer patients were selected from the specimen repository, and normal pancreatic epithelial tissues adjacent to cancer were taken. RNA was extracted, and pooled in equal quantities. Reverse transcription of cDNAs were used as a control. As shown in the Figure 3(A), PSMC2 mRNA was highly expressed in the three pancreatic cancer cell lines tested, and the average of ΔCt values were 6.07, 5.12, 5.96 respectively. As *PSMC2* is expressed most highly in SW1990 cells, these cells were selected for subsequent *PSMC2*-knockdown experiments. Effective knockdown of *PSMC2* by shRNA was confirmed in SW1990 cells using immunoblotting (Figure 3(B)). After infection with recombinant lentiviruses, >80% of SW1990 cells were shown to express GFP under a fluorescence microscope (Figure 3(C)). Upon lentiviral infection, *PSMC2* mRNA was significantly reduced in SW1990 cells compared with cells infected with control shRNA (Figure 3(D)).

### 3.4 Knockdown of PSMC2 inhibited pancreatic cancer cell proliferation

**Figure 4:**
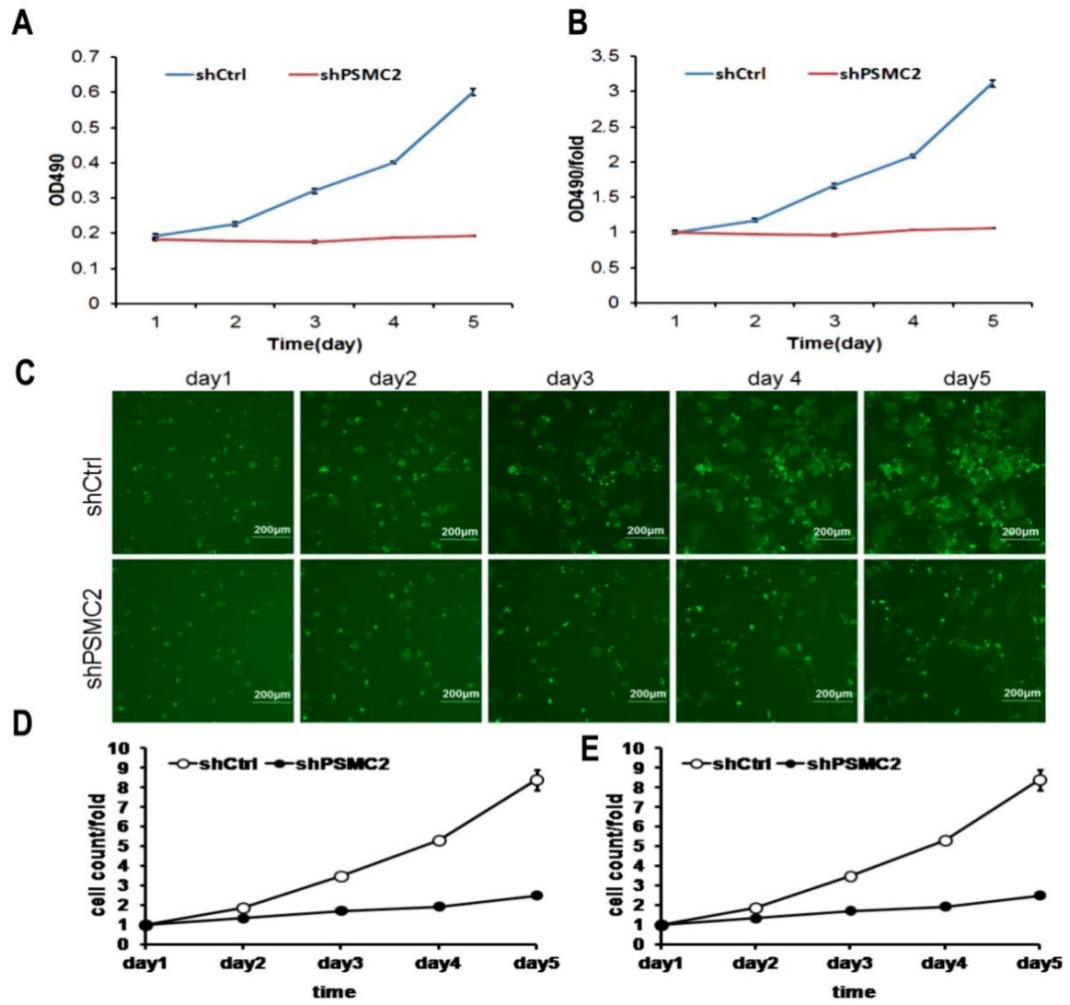
*PSMC2* knockdown is associated with reduced proliferation in SW1990 cells. Both Celigo cell counting and MTT absorbance were performed to measure the rate of cell proliferation. (A, B), cell growth curves were plotted using MTT absorbance. (C), fluorescent photomicrographs were taken for cells expressing green fluorescence protein at the indicated times. (D, E), cell growth was depicted every day for 5 days calculated using algorithms and the raw image data (shCtrl vs shPSMC2 , p<0.05).

MTT assays were performed to assess proliferation in *PSMC2* knockdown SW1990 cells. Growth rate of sh*PSMC2* SW1990 cells was much slower than that of the shCtrl cells (*p*<0.01, Figures 4(A,B)). Similarly, the growth curve counted and generated by Nexcelom Celigo Image Cytometer showed that clonogenic survival was significantly decreased following knock down of *PSMC2* in SW1990 cells by shRNA (Figures 4C-E). These results suggested that *PSMC2* may act as an oncogene that increases proliferation of pancreatic cancer cells.

### 3.5 Knockdown of *PSMC2* increased pancreatic cancer cell apoptosis

**Figure 5:**
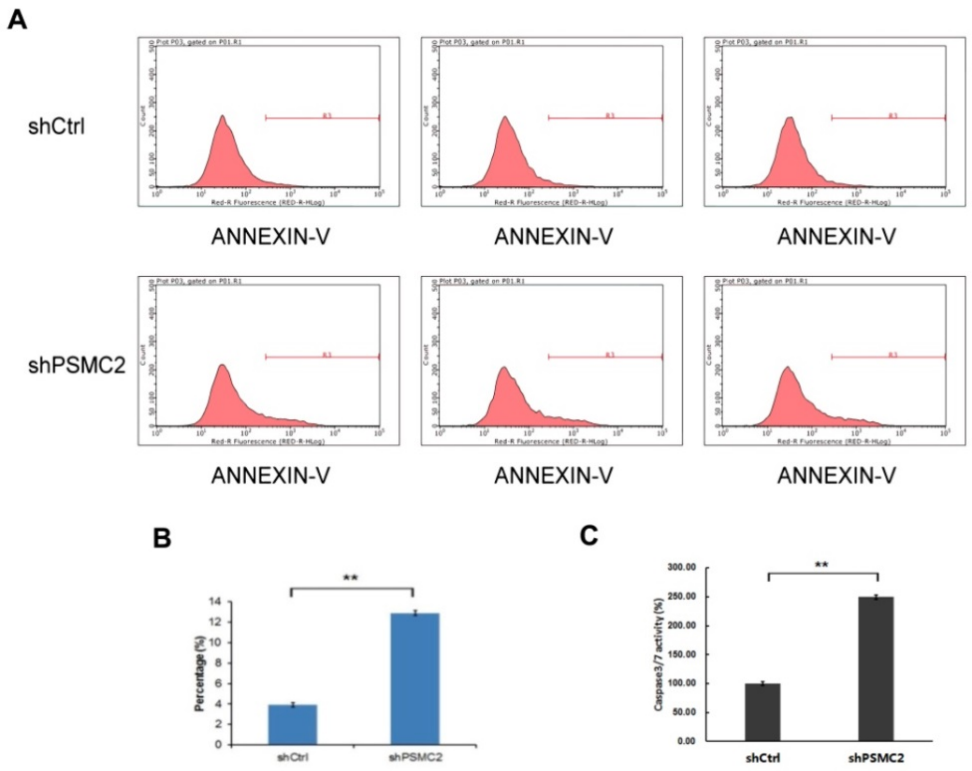
*PSMC2* knockdown is associated with elevated apoptosis in SW1990 cells. (A, B), cell death was determined by Annexin V staining followed by flow cytometric analysis.(C), quantification of percentage of apoptotic cells, as measured by caspase 3/7 activity. (**p<0.01).

Apoptosis of SW1990 cells was measured by FACS analysis of Annexin-V and Caspase 3/7 array. Knockdown of *PSMC2* in SW1990 cells increased apoptosis from 4% to 13.12% (Figure 5(A,B)). To provide further evidence for a role of PSMC2 in apoptosis, sh*PSMC2* cells or shCtrl cells were cultured on a 96-well plate and subjected to Caspase 3/7 activity analysis. Consistent with the results from Annexin V staining, significantly higher levels of caspase3/7 activities were present in cells expressing sh*PSMC2,* compared with those expressing shCtrl (Figure 5(C)). Together, these results showed that PSMC2 was important in the regulation of cell viability.

## 4. Discussion

The ubiquitin-proteasome system (UPS) is a major mechanism for selective protein degradation in eukaryotic cells 14.The UPS contains two major steps. The first step is the covalent attachment of ubiquitin(s) to a protein substrate, a process called ubiquitination, and the second is the degradation by the 26S proteasome 15. The 26S proteasome is the major nonlysosomal protease in eukaryotic cells and is responsible for the degradation of all short-lived proteins and 70% to 90% of all long-lived proteins. Thus, it is not surprising that the 26S proteasome plays a critical role in many cellular functions, and, in particular, it is closely involved in many regulatory pathways, including cell proliferation and apoptosis. Indeed, the26S proteasome has been viewed as a potential target for the treatment of human cancers**.**

Recent studies showed that the proteasome plays an important role in the proliferation and apoptosis of the pancreatic cancer. Two decades ago, Theodore P*et al*. had already showed that PSI, a peptide aldehyde inhibitor of the 26S proteasome, effectively induces apoptosis in BxPC-3 human pancreatic cancer cells 16. Wang *et al*. showed that quick loss of PSMD11 (26S proteasome non-ATPase regulatory subunit 11) induced rapid or acute apoptosis in pancreatic cancer cells 17. Furthermore, regulation of an evolutionarily conserved RNA polymerase II-associated factor 1(PAF1) by UPS was involved in pancreatic oncogenesis. In poorly differentiated pancreatic cancer, elevated PAF1 levels were associated with downregulation of proteasomal degradation 18.

PSMC2 is part of the 19S regulatory complex of the 26S proteasome and its expression is essential for 19S and 26S proteasome assembly. In a synthetic lethal screen by comparative genomic approach, *PSMC2* was identified to have genetic interaction with *ASPSCR1* 11. This gene pair involves components of the proteasome and is related to chromosome stability. Nijhawan *et al*. defined *PSMC2* as a top one in CYCLOPS genes, which represent a distinct class of cancer-specific liabilities resulting from genome instability 8. The study indicated that the frequency of partial genomic loss of *PSMC2* was 0.10 among 3131 cancers across a wide diversity of cancer types, rendering a high dependence of cancer cells on the remaining *PSMC2*. This result further suggested PSMC2 could serve as a potential target for cancer treatment. Indeed, the authors showed that PSMC2 suppression decreases ovarian cancer cell proliferation and its expression also correlated with pancreatic cancer cell proliferation 8. PSMC2 also expressed highly in osteosarcoma samples, as determined by tissue microarrays analysis. Suppression of *PSMC2* reduces cell proliferation and increases apoptosis, which is consistent with it acting as an oncogene for osteosarcoma 10. However, whether *PSMC2* carries out the similar functions in pancreatic cancer remains unexplored.

Here, we evaluated PSMC2 expression in pancreatic adenocarcinoma using IHC for the first time and conducted cDNA microarray analysis by using the Oncomine database to explore gene expression of *PSMC2* in pancreatic adenocarcinoma tissues. These findings suggested that *PSMC2* may work oncogenic function. Then, we demonstrated that *PSMC2* expression was critical for pancreatic cancer cell survival. First, we showed that *PSMC2* was ubiquitously expressed in SW1990, PANC-1 and AsPC-1pancreatic cancer cell lines. Using RNA interference, we found that *PSMC2* was necessary for proliferation and also suppressed apoptosis of pancreatic cancer cells.

The molecular mechanisms underlying *PSMC2* in the process of tumorigenesis are not clear. PSMC2 likely drives osteosarcoma via its regulation of cancer-related genes, including *ITGA6*, *FN1*, *CCND1*, *CCNE2* and *TGFβR2* 10. Excess PSMC2 in normal cells resides in a complex with PSMC1, PSMD2, and PSMD5, which acts as a reservoir protecting cells from the anti-proliferative effect associated with *PSMC2* suppression. Cells harboring partial *PSMC2* copy number loss lack this complex and die upon PSMC2 suppression 8. Besides its proteolytic roles, the 26S proteasome reportedly regulates transcription and promoting sites of active chromatin 19. In addition, the 19S subunit of the 26S proteasome may also regulate the spreading of heterochromatin 20. However, these non-canonical functions of the 26S proteasome in transcriptional regulation are still largely debatable. Further studies will be necessary to functionally validate the role of PSMC2 in tumorigenesis *in vivo* and to uncover the underlying molecular mechanisms.

In conclusion, our study for the first time revealed an important function of PSMC2 in mediating proliferation and apoptosis in pancreatic cancer, and suggested that PSMC2 might serve as a potential therapeutic target for the treatment of pancreatic cancer. Understanding the precise role of PSMC2 in pancreatic cancer pathogenesis will be critical and might facilitate the development of anti-tumor therapies against PSMC2.

## Figures and Tables

**Table 1:** Clinicopathological characteristics and the status of PSMC2 expression in pancreatic adenocarcinoma.

Factors	PSMC2 expression		
	negative	positive	* P-*value
Age (years)			
≤60	4	9	
>60	8	19	0.941
Gender			
Female	5	8	
Male	7	20	0.418
Place			
Head	9	21	
Tail	3	7	1.000
Vascular invasion			
Yes	2	18	
No	10	10	0.014
Lymphatic invasion			
Yes	5	24	
No	7	4	0.008
Neruo invasion			
Yes	7	22	
No	5	6	0.254
Stats			
I-II	8	4	
III	4	24	0.002

## Abbreviations

PSMC2: Proteasome 26S subunit ATPase 2
qRT-PCR: quantitative RT-PCR
shRNA: short hairpin RNA
FACS: fluorescence-activated cell sorting
OS: overall survival
CYCLOPS: copy number alterations yielding cancer liabilities owing to partial loss
IHC: Immunohistochemistry
GFP: green fluorescent protein
RIPA: radioimmunoprecipitation assay
PVDF: polyvinylidene difluoride
siRNA: Small interference RNA
H&E: Hematoxylin and eosin
UPS: ubiquitin-proteasome system
PAF1: polymerase II-associated factor 1
